# Error-related potentials during multitasking involving sensorimotor control: an ERP and offline decoding study for brain-computer interface

**DOI:** 10.3389/fnhum.2025.1516721

**Published:** 2025-01-28

**Authors:** Masaki Yasuhara, Isao Nambu

**Affiliations:** Graduate School of Engineering, Nagaoka University of Technology, Nagaoka, Japan

**Keywords:** error-related potentials, error-related negativity, EEG, dual-task, human-computer interaction, brain-computer interface, error monitoring

## Abstract

Humans achieve efficient behaviors by perceiving and responding to errors. Error-related potentials (ErrPs) are electrophysiological responses that occur upon perceiving errors. Leveraging ErrPs to improve the accuracy of brain-computer interfaces (BCIs), utilizing the brain's natural error-detection processes to enhance system performance, has been proposed. However, the influence of external and contextual factors on the detectability of ErrPs remains poorly understood, especially in multitasking scenarios involving both BCI operations and sensorimotor control. Herein, we hypothesized that the difficulty in sensorimotor control would lead to the dispersion of neural resources in multitasking, resulting in a reduction in ErrP features. To examine this, we conducted an experiment in which participants were instructed to keep a ball within a designated area on a board, while simultaneously attempting to control a cursor on a display through motor imagery. The BCI provided error feedback with a random probability of 30%. Three scenarios–without a ball (single-task), lightweight ball (easy-task), and heavyweight ball (hard-task)–were used for the characterization of ErrPs based on the difficulty of sensorimotor control. In addition, to examine the impact of multitasking on ErrP-BCI performance, we analyzed single-trial classification accuracy offline. Contrary to our hypothesis, varying the difficulty of sensorimotor control did not result in significant changes in ErrP features. However, multitasking significantly affected ErrP classification accuracy. *Post-hoc* analyses revealed that the classifier trained on single-task ErrPs exhibited reduced accuracy under hard-task scenarios. To our knowledge, this study is the first to investigate how ErrPs are modulated in a multitasking environment involving both sensorimotor control and BCI operation in an offline framework. Although the ErrP features remained unchanged, the observed variation in accuracy suggests the need to design classifiers that account for task load even before implementing a real-time ErrP-based BCI.

## 1 Introduction

Error perception is crucial for achieving efficient behaviors. For instance, when reaching for an object, even a slight deviation in hand position leads to errors. Precise action is achieved through feedback strategies that respond to errors. Error signals can be observed as electrophysiological responses measurable by electroencephalography (EEG) and are referred to as error-related potentials (ErrPs). Typically, approximately 250 ms after erroneous feedback, a negative peak known as error-related negativity (ERN) is observed, followed by a positive potential known as error positivity (Pe) (Ferrez and Millán, 2005). Ferrez and Del R. Millan (2008) integrated error detection and correction strategies into a brain-computer interface (BCI). Traditionally, BCIs decode EEG signals such as P300 and motor imagery to generate commands for communication aids or robotic device control (Rashid et al., 2020): users can input text without physical movement (Pan et al., 2022), or control a wheelchair directly via brain signals (Naser and Bhattacharya, 2023). The incorporation of ErrP into BCIs enhances system performance by facilitating error correction strategies (Chavarriaga et al., 2014; Zeyl et al., 2016; Cruz et al., 2018; Kim et al., 2019; Parashiva and Vinod, 2022). Hence, the development of ErrP-based BCIs (ErrP-BCIs) that integrate error detection and correction strategies is critical for enhancing system usability.

An important question is whether the ErrP-BCIs can function effectively in multitasking environments. Although most studies on BCI have been conducted in controlled laboratory settings, recent studies have focused on how these systems operate in more realistic multitasking scenarios that reflect everyday life (Huang et al., 2020). A study on the P300 speller, a widely used BCI, demonstrated that increasing cognitive load slows neural responses to stimuli, resulting in decreased system performance (Ke et al., 2016). As mental workload increases, cognitive resources become more dispersed, leading to a reduction in P300 amplitude (Käthner et al., 2014).

ErrP modulation has been assessed in the context of the availability of cognitive resources. Sleep deprivation has been demonstrated to slow down reaction times and reduce Pe amplitudes (Boardman et al., 2024). ERN amplitude is significantly correlated with sustained attention, where reduced attention leads to a decrease in ERN amplitude (Xiao et al., 2015). Similarly, attention bias correction lowers attention and diminishes ERN amplitude (Nelson et al., 2017). In contrast, task evaluation in flanker tasks increases attention, resulting in greater amplitudes of ERN and Pe (Grützmann et al., 2014). These results indicate that ErrPs are affected by attentional resources and cognitive load. Studies on ErrP modulation under multitasking cognitive load have revealed that increasing cognitive demands reduces ERN amplitude (Tanaka et al., 2005; Klawohn et al., 2016). Studies on multitasking have primarily focused on cognitive load.

However, to our knowledge, ErrP modulation during sensorimotor control remains unexplored. In real-life scenarios, such as operating a navigation system while driving, performing sensorimotor tasks along with interfacial operations is common. Extending physical control through simultaneous interface operations during sensorimotor tasks represents an ideal application of BCI (Penaloza and Nishio, 2018; Eden et al., 2022). Studies have been conducted to address the challenges associated with multitasking involving both sensorimotor control and BCI operation (Penaloza and Nishio, 2018; Bashford et al., 2018). Investigating ErrPs related to BCI operation during sensorimotor control is crucial for determining the applicability of ErrP-BCIs.

This study aimed to investigate whether ErrPs generated during BCI operations are affected by multitasking with sensorimotor control. Similar to previous studies on cognitive load (Tanaka et al., 2005; Klawohn et al., 2016), we hypothesized that ErrPs would fluctuate because of the distribution of neural resources caused by multitasking during sensorimotor control. However, Iwane et al. (2021) showed that errors can still be detected using the same classifier despite such fluctuations; accordingly, we expected that errors in different scenarios could also be detected using the same classifier during multitasking. To test this hypothesis, we designed an experimental paradigm that intentionally induced BCI errors during multitasking using sensorimotor control. To replicate real-life scenarios, participants were asked to control the BCI while carrying an object with both hands. Specifically, they attempted to keep a ball steady on a board while simultaneously performing motor imagery EEG to move a cursor on a screen in an instructed direction. The cursor moved in the opposite direction in 30% of the trials, regardless of EEG decoding. Varying the weight of the ball, we examined the characteristics of ErrPs at various sensorimotor control levels.

## 2 Materials and methods

### 2.1 Participants

A total of 28 individuals (two females, mean age 23.75 ± 3.27 years) participated in the study. Three participants whose trials yielded more than 80% invalid results (see below: data analysis) were excluded from the analysis. Therefore, data from 25 participants were used in the final analysis. The experimental protocol was approved by the local ethics committee of Nagaoka University of Technology (Number 2023-03-03), and written informed consent was obtained from all participants prior to the experiment. This study was conducted in accordance with the principles of the Declaration of Helsinki.

### 2.2 Experimental setup

**Figure 1:**
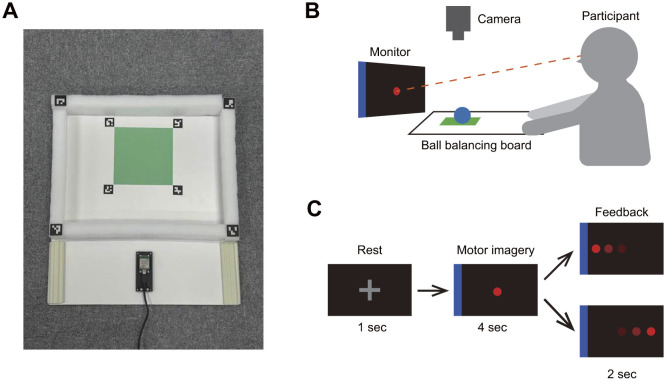
The experimental setup. **(A)** The ball-balancing board had walls, a green area, and ArUco markers. **(B)** Participants were seated and held the ball-balancing board with both hands. A monitor was placed in front of the board to provide cues and feedback. Throughout the session, participants performed the ball-balancing-board task, keeping the ball within the green area. Participants were instructed to keep their gaze fixed on the center of the monitor, and could only focus on the ball if the ball moved outside the green area. **(C)** The monitor displayed rest cues, motor imagery cues, and feedback. During the motor imagery phase, a blue rectangle appeared on either the left or right side of the screen, while a red cursor was located at the center. Participants were instructed to imagine clenching the hand on the side of the blue line for 4 s. After this, feedback was provided through the movement of the cursor.

An experimental environment was constructed to simultaneously measure EEG and electromyography (EMG) signals while participants performed sensorimotor control and BCI tasks. The sensorimotor control task was a modified version of the ball-balancing-board task used by Penaloza and Nishio (2018). In the modified task, participants were required to keep a ball within a designated area on a custom-made ball-balancing board (width: 450 mm; height: 600 mm) featuring a green area, walls, and ArUco markers (Figure 1A). A camera (C980; Logicool Co.) was positioned to capture the entire board to detect both the ArUco markers and the position of the ball (Figure 1B). A monitor was placed near the board, allowing participants to view the board and the monitor simultaneously. To vary the difficulty of the sensorimotor control task, three scenarios were tested: without a ball, with a light rubber ball (diameter: 49 mm, weight: 55 g; D7222; Danno Works, Osaka, Japan), and with a heavy metal ball (diameter: 40 mm, weight: 265 g; SUS304).

EEG data, EMG data, and trigger information were streamed using the Lab Streaming Layer^1^ (LSL) and recorded using LabRecorder software. EEG data were streamed using App-Biosemi software, and EMG data were streamed using custom-made software based on the Trigno Software Development Kit. The monitor was controlled by PsychoPy (Peirce et al., 2019), which streams trigger information to the LSL during stimulus presentation changes. The camera was controlled using OpenCV integrated into PsychoPy, and the video was continuously recorded at 30 frames per second in a format that allowed separation by trial.

### 2.3 Experimental design

The participants were required to multitask, involving both sensorimotor control and motor-imagery-based BCI operation. While performing the ball-balancing task, the participants attempted to move the cursor in the instructed direction based on imagined hand movements. The gaze was fixed at the center of the screen, and attention was allowed to shift to the ball only when it moved out of the green area. Prior to data collection, a familiarization session was conducted for approximately 5 min, during which participants practiced keeping the ball within the green area while focusing on the monitor. During the familiarization session, the experimenter ensured that the participants fully understood the experimental process. Figure 1C shows a cue diagram. Initially, a fixation point was displayed on the monitor for 1 s, followed by the presentation of a red cursor and a blue rectangle for 4 s. The blue rectangle appeared randomly on either the left or right side of the screen, and the task was to move the red cursor toward the blue rectangle using motor imagery. Prior to the experiment, event-related desynchronization was explained to the participants, emphasizing that the cursor movement reflected neural activity related to imagined hand movements. After the motor imagery phase, feedback was provided by moving the red cursor to either the left or the right side. The actual feedback was programmed to include a 30% error rate, leading to the cursor moving in the wrong direction independent of the EEG signals. This error rate was selected based on previous ErrP studies (Kim et al., 2019; Usama et al., 2020; Iwane et al., 2023).

We designed three sessions: a single-task session (without a ball), an easy-task session (lightweight ball), and a hard-task session (heavyweight ball). Each session consisted of three blocks with 40 consecutive trials per block. This resulted in 84 correct and 36 erroneous feedback trials for each scenario (single, easy, and hard tasks). To minimize the effects of fatigue (Xiao et al., 2015), participants were given a 3-min break between blocks and a 5-min break between sessions. Participants were allowed to take longer breaks if needed. The first and second sessions involved multitasking scenarios (easy and hard tasks); the order of the sessions was randomized. The third session, which consisting of the single-task scenario, served as a control experiment to examine the effects of multitasking. Additionally, to investigate temporal adaptations, such as fatigue or learning effects, 15 participants completed an extra single-task session before the multitasking sessions. Of these 15 participants, two were excluded from the analysis, resulting in data from 13 participants that were used to analyze the effects of temporal adaptation.

### 2.4 Questionnaire

A two-section questionnaire was administered for each block. One section assessed the difficulty of the ball-balancing board task on a 5-point scale to verify whether the task difficulty was appropriately set. Difficulty was assessed only for easy and hard tasks. The other section evaluated the reliability of kinesthetic motor imagery on a 5-point scale to determine whether multitasking interfered with motor imagery. A motor imagery reliability assessment was conducted during all sessions using a questionnaire adapted from the Japanese version (Nakano et al., 2018) of the motor imagery scale developed by Malouin et al. (2007). The data collected after each block were averaged for each session and participant.

### 2.5 EEG acquisition

Continuous EEG data were recorded using the Active Two system (Biosemi, Amsterdam, Netherlands). A 32-channel cap utilizing the international 10-20 layout was used with the common mode sense and the driven right leg electrodes placed on the cap. Additional electrodes were placed on the earlobes, and vertical and horizontal electrooculogram (EOG) electrodes were attached following the method described by Croft and Barry (2000). The offset voltage was maintained at ± 25 mV. Continuous EEG data were streamed to the LSL at a sampling rate of 2,048 Hz.

### 2.6 EMG acquisition

EMG was recorded to assist in trial rejection. Surface EMG activity was recorded in the extensor digitorum and flexor digitorum superficialis of both arms. Continuous EMG data were measured using a Trigno wireless system (Delsys, Boston, MA, USA) and streamed to the LSL at a sampling rate of 2 kHz using custom-made software based on the Trigno Software Development Kit.

### 2.7 Data analysis

Python (version 3.12.2) was used for all data analyses, including EEG and EMG signal processing, and ball position detection. MATLAB (version R2024a), EEGLAB (version 2024.1), and ERPLAB (version 12.00) were used to quantify ErrPs.

#### 2.7.1 Error-related potentials

EEG data were pre-processed and analyzed using MNE-python 1.7.1 (Gramfort, 2013). Continuous EEG data were first resampled to 500 Hz and referenced to the average earlobe electrodes. A bandpass filter of 1-40 Hz was applied, and bad channels were marked for interpolation. An independent component analysis (ICA) was performed to remove artifacts related to eye movement and muscle activity. The components to be removed were selected through visual inspection with reference to the recorded EOG data and ICLabel-based classification (Pion-Tonachini et al., 2019; Li et al., 2022). The marked bad channels were interpolated using spherical spline interpolation. The ICA-processed EEG data were segmented into epochs ranging from -250 to 1,000 ms, time-locked to the feedback onset. Epochs with EEG amplitudes exceeding 150 μV were rejected to minimize contamination from artifacts. Epochs were created using the remaining valid trials. Baseline correction was applied with a range of -250 to 0 ms relative to the feedback onset. ERN and Pe were analyzed within time windows of 150–300 ms and 250–550 ms, respectively. ErrPs were computed separately for error and correct feedback trials. Visual inspection of the grand averaged ErrPs, along with previous information regarding motor imagery-based BCI-related ErrPs (Usama et al., 2020), was used to define the time windows for quantification. For each component, the peak amplitude and peak latency were quantified using the ERPLAB toolbox (Lopez-Calderon and Luck, 2014). This toolbox offers advanced functionalities for ERP analysis, including algorithms for local peak detection (Luck, 2014). Time-frequency domain features were extracted to further investigate neural activities associated with error processing. Event-related spectral perturbations (ERSPs) were computed from the epoched data to capture changes in the spectral power related to error events. ERSPs were calculated as dB values, representing the log ratio of power during the epoch relative to the baseline period. To prevent contamination from task-related brain activity (Pfurtscheller and Lopes Da Silva, 1999), data from a 3-min resting-state EEG recorded with eyes open prior to the experiment was used as the baseline.

#### 2.7.2 EMG power

EMG data were preprocessed with a 30-200 Hz bandpass filter and a 50 Hz notch filter to remove power-line noise. The data were then subjected to full-wave rectification and smoothened with a 5 Hz low-pass filter. Unintended muscle movements during motor imagery tasks can induce ErrPs that differ from those associated with BCI operations. Therefore, trials with significant changes in muscle power were classified as erroneous and excluded from the analysis. The exclusion threshold was determined by calculating the average EMG power during the resting period. If the peak EMG power during the motor imagery exceeded twice the average rest-period power, the trial was excluded. The threshold was established based on the findings of the pilot experiment.

#### 2.7.3 Ball position detection

The video data were separated for each trial, and the board position, along the green area, was detected using OpenCV and ArUco markers. The center coordinates of the ball were estimated by detecting the circular edges using the Hough transform in OpenCV. In this experiment, two types of errors occurred: BCI-related errors and ball-balancing board-related errors. To exclude trials with ErrPs induced by ball-balancing-board errors, trials in which the center coordinates of the ball moved outside the green area were identified. Any trial in which the ball was outside the green area, was excluded from the analysis.

### 2.8 Classification

Pre-processing and classification were performed as per a review by Yasemin et al. (2023). First, the ICA-processed EEG were referenced using a common average reference and were filtered using a low-pass filter of 10 Hz. The data were then resampled to 60 Hz, and a time window of 200–800 ms relative to the feedback onset was extracted. An overlap window average was used to reduce the number of features. The classification was performed using shrinkage linear discriminant analysis implemented in Scikit-learn (Pedregosa et al., 2011). The classifier categorized ErrPs as either erroneous or correct trials. The performance was evaluated using balanced accuracy, which is used in ErrP-BCI evaluations to account for imbalances between the number of erroneous and correct trials (Yasemin et al., 2023; Iwane et al., 2023). The balanced accuracy was calculated as the average sensitivity (true-positive rate) and specificity (true-negative rate). In addition, transfer learning was applied to investigate whether ErrPs under multitasking scenarios could be classified using a model trained on single-task data. Specifically, a classifier was trained on ErrPs from the single-task scenario and then tested on multitasking data to assess balanced accuracy in more complex environments. Herein, we refer to this as transfer (easy) for the easy-task scenario and transfer (hard) for the hard-task scenario in transfer learning. To determine whether the mean-balanced accuracy exceeded chance, a permutation test was conducted for each task. The class labels were shuffled, and the grand-averaged balanced accuracy across participants was calculated 1000 times. The chance level was defined as the 95th percentile of the resulting distribution.

### 2.9 Statistics

All statistical analyses of the questionnaire scores, EEG responses, and classification accuracies were performed using R (version 4.4.3) with the ez (version 4.4-0) and rstatix (version 0.7.2) packages and MNE-python for a cluster-based permutation test. The threshold for statistical significance was set at *p* = 0.05.

#### 2.9.1 Questionnaire

A paired t-test was conducted to examine whether the participants perceived a difference in difficulty between the easy and hard multitasking scenarios. To investigate whether multitasking affected the reliability of motor imagery, a one-way repeated-measures analysis of variance (ANOVA) was performed with task scenarios (single, easy, and hard tasks) as a within-subject factor. If the assumption of sphericity was violated, the Greenhouse-Geisser correction was applied. *Post-hoc* tests with Benjamini-Hochberg correction (Benjamini and Hochberg, 1995) for multiple comparisons were applied when significant effects were observed.

#### 2.9.2 Error-related potentials

To investigate the effects of multitasking on ErrPs, a two-way repeated-measures ANOVA was conducted on the peak amplitude and peak latency of ERN and Pe, with task scenarios (single, easy, and hard tasks) and feedback type (erroneous and correct) as within-subject factors. Based on previous studies, repeated measures ANOVA was chosen to investigate modulations in ErrPs (Tanaka et al., 2005; Klawohn et al., 2016; Usama et al., 2020). The Greenhouse-Geisser correction was applied when the assumption of sphericity was violated. Additionally, to examine the effects of multitasking in the frequency domain, a cluster-based permutation test was performed using 2,000 permutations (Maris and Oostenveld, 2007; Sassenhagen and Draschkow, 2019). Previous studies on ErrP typically focused on the FCz or Cz channels, where the peak amplitudes tended to be maximal (Ferrez and Millán, 2005; Ferrez and Del R. Millan, 2008; Gehring et al., 2011). As the 32-channel EEG cap used herein did not include the FCz, statistical analyses were conducted using the Cz channel.

#### 2.9.3 Classification

To investigate the effects of multitasking on ErrP classification, a one-way repeated-measures ANOVA was conducted with the classification scenarios (single task, easy task, hard task, transfer easy, and transfer hard) as within-subject factors. The Greenhouse-Geisser correction was applied when the assumption of sphericity was violated. For significant effects, *post-hoc* tests with Benjamini-Hochberg correction for multiple comparisons were performed.

#### 2.9.4 Temporal adaptation effect

Fatigue alters the characteristics of ErrPs (Xiao et al., 2015). To investigate temporal adaptation effects, such as fatigue or learning effects, a statistical analysis was conducted on the differences between the first and last single-task sessions. The same methods used in the multitasking analysis were used to assess the questionnaires, ErrPs, and classification performance.

## 3 Results

Owing to EEG signal quality, errors in EMG power, and ball position detection, 22.6% of all trials were excluded from analyses. The success rates for the ball-balancing task were 95.0% (variance: 30.7) for the easy-task and 84.6% (variance: 138.5) for the hard-task. Additionally, the removal rates due to EMG-related errors were 18.7% for the single-task, 16.1% for the easy-task, and 19.2% for the hard-task.

### 3.1 Questionnaire

**Figure 2:**
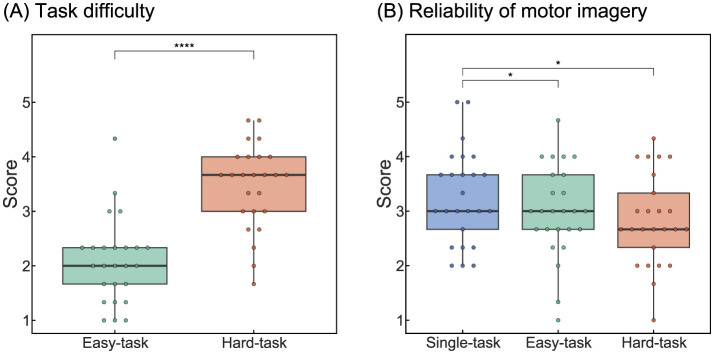
Results of the questionnaire analysis. **(A)** Task difficulty of multitasking scenarios. **(B)** Reliability of kinesthetic motor imagery. Statistical significance: ^*^p < 0.05,^****^p < 0.0001.

We first investigated whether the participants perceived a difference in difficulty between the easy and hard tasks using questionnaire data (Figure 2A). The grand-averaged difficulty scores across participants were 2.08 for the easy tasks and 3.47 for the hard tasks. A paired t-test revealed a significant difference between the two scenarios (*t*_(24)_ = −8.45, *p* < 0.0001), indicating that participants clearly perceived the difference in task difficulty and that the task difficulty was appropriately set by these scenarios.

The grand-averaged scores for the reliability of kinesthetic motor imagery were 3.27, 3.00, and 2.84 for the single, easy, and hard tasks, respectively (Figure 2B). The ANOVA revealed a significant main effect of task scenarios (*F*_(2, 48)_ = 4.63, *p* < 0.05). *Post-hoc* tests showed significant differences between the single- and easy-task scenarios (*t*_(24)_ = 2.28, *p.adj* < 0.05) and between the single- and hard-task scenarios (*t*_(24)_ = 2.74, *p.adj* < 0.05). These results suggested that multitasking interfered with the reliability of motor imagery.

In summary, the findings indicated that the participants experienced interference in their motor imagery during BCI operation with multitasking. Although participants perceived a difference in difficulty between the two multitasking scenarios, task difficulty did not appear to affect kinesthetic motor imagery reliability.

### 3.2 Error-related potentials

**Figure 3:**
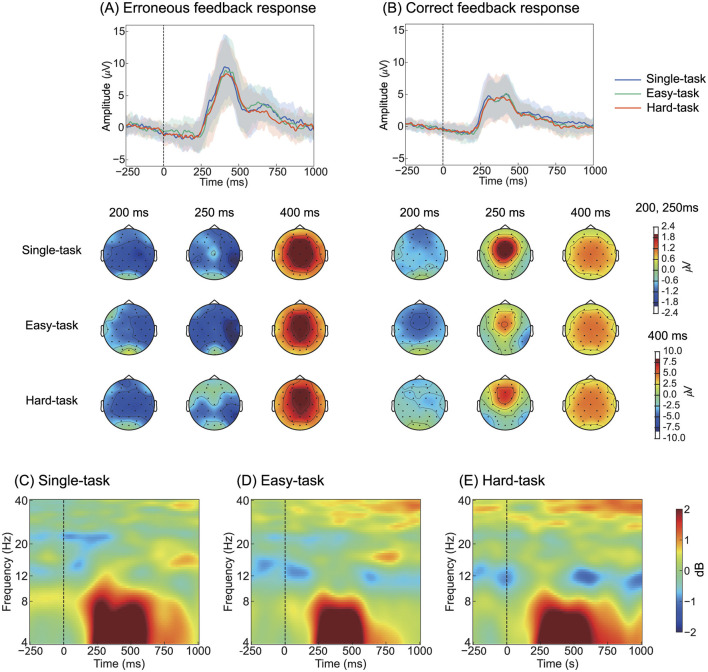
Grand-averaged ErrPs at Cz with topographic distribution maps and event-related spectral perturbations at Cz. **(A)** ErrPs responses to erroneous feedback and **(B)** correct feedback. The shaded area around the line represents the standard deviation across all participants. The three time points shown in the topographic maps correspond to 250 ms and 400 ms as defined by Ferrez and Millán (2005), with an additional 200 ms selected based on visual inspection. Color represents amplitudes in μV. **(C–E)** Event-related spectral perturbations for erroneous feedback trials, computed using the eyes-open condition recorded prior to the experiment as the baseline. Color represents amplitude in dB. ErrP, Error-related potentials.

To investigate the effect of multitasking on ErrPs, we compared ErrPs across task scenarios in both time and frequency domains (Figure 3). During erroneous feedback, ERN was observed at approximately 212 ms after feedback, followed by Pe at approximately 418 ms (Figure 3A, Table 1). During correct feedback, correct-related negativity (CRN) was observed at approximately 194 ms after feedback, followed by a positive peak at approximately 386 ms (Figure 3B, Table 1). Topographical maps showed that ERN was distributed across all scalp electrodes, whereas Pe was primarily observed in the frontal-central region (Figures 3A, B). In the frequency domain, ErrP components were detected in the 4-8 Hz range across all tasks (Figures 3C–E).

**Table 1:** Summary of peak amplitude and peak latency of ERN and Pe across all participants for the task scenarios (single, easy, and hard tasks).

		**Peak amplitude (**μV**)**		**Peak latency (ms)**	
		**ERN**	**Pe**	**ERN**	**Pe**
Single-task	Erroneous	−3.78 ± 2.08	11.4 ± 4.72	211 ± 42.3	412 ± 30.8
	Correct	−2.01 ± 1.42	6.68 ± 3.11	188 ± 28.1	387 ± 71.9
Easy-task	Erroneous	−3.18 ± 1.99	11.0 ± 5.46	217 ± 37.2	422 ± 51.2
	Correct	−2.08 ± 1.41	6.58 ± 3.13	194 ± 25.3	389 ± 64.5
Hard-task	Erroneous	−3.62 ± 2.38	10.9 ± 4.72	207 ± 43.7	419 ± 55.4
	Correct	−2.01 ± 1.04	6.89 ± 3.47	199 ± 37.2	381 ± 66.2

Values are presented as the mean ± standard deviation. ERN, error-related negativity; Pe, error positivity.

ANOVA for the peak amplitude of ERN revealed a significant main effect of the feedback type (*F*_(1, 24)_ = 26.4, *p* < 0.05). Similarly, ANOVA for the peak latency of ERN showed a significant main effect of the feedback type (*F*_(124)_ = 8.35, *p* < 0.05). For the peak amplitude of Pe, ANOVA revealed a main effect of the feedback type (*F*_(1, 24)_ = 72.2, *p* < 0.05); similarly, for the peak latency of Pe, a significant main effect of the feedback type was observed (*F*_(1, 24)_ = 13.5, *p* < 0.05). Detailed statistical results are provided in Supplementary Table 1. These results indicate that ERN and CRN differ, with erroneous feedback showing greater amplitude and longer latency. The cluster-based permutation test for the ERSP analysis did not reveal any significant clusters.

Overall, both time- and frequency-domain analyses suggested that electrophysiological responses were not influenced by multitasking.

### 3.3 Classification

**Figure 4:**
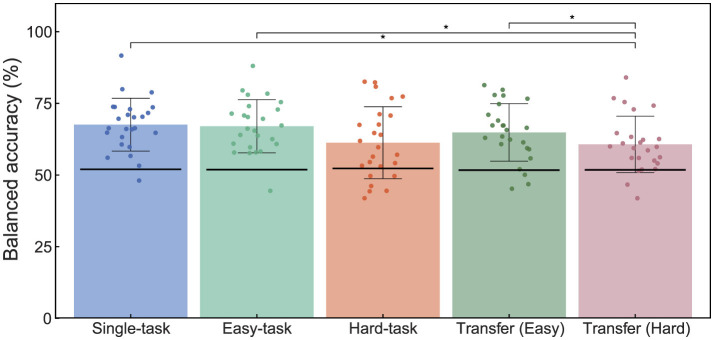
Effects of simultaneous sensorimotor control on ErrP classification during BCI operation. The bar graphs labeled Single-task, Easy-task, and Hard-task represent the classification performance within their respective scenarios. Transfer (Easy) and Transfer (Hard) show the classification results when the classifier was trained on Single-task ErrPs and tested on data from Easy- and Hard-task scenarios, respectively. Each dot indicates the accuracy for each participant. The black lines on each bar graph indicate the chance level calculated using the permutation test. Statistical significance: ^*^p < 0.05. ErrP, Error-related potentials; BCI, brain-computer interface.

Figure 4 summarizes the accuracy of the ErrP classification evaluated using a shrinkage linear discriminant analysis. The grand-averaged accuracies were 67.6%, 67.0%, 61.3%, 64.9%, and 60.7% for the single, easy, hard, transfer (easy), and transfer (hard) tasks, respectively. The results of the permutation test demonstrated that all classifiers performed above the chance level, indicating that errors can be detected by the classifier even in multitasking scenarios.

The results of the one-way repeated measures ANOVA showed a significant main effect (*F*_(4, 96)_ = 4.74, *p* < 0.05). *Post-hoc* tests revealed significant differences between the single-task and transfer-hard scenarios (*t*(24) = 3.25, *p.adj* < 0.05), easy-task and transfer-hard scenarios (*t*(24) = 3.49, *p*.*adj* < 0.05), and transfer-easy and transfer-hard scenarios (*t*_(24)_ = 2.62, *p.adj* < 0.05).

These results indicate that while multitasking affects ErrP classification accuracy, training the classifier on data from multitasking scenarios can yield a classification performance comparable to that of single-task scenarios. Conversely, if the classifier is trained exclusively on data from a single-task scenario, the accuracy may decrease for multitasking scenarios.

### 3.4 Temporal adaptation effect

**Figure 5:**
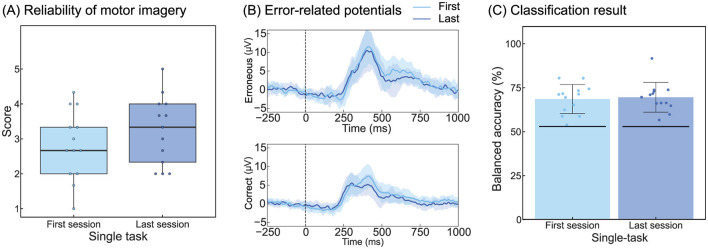
Effects of temporal adaptation on participants and the classifier. **(A)** Results of the questionnaire analysis for reliability of kinesthetic motor imagery. The format is the same as that in Figure 2B. **(B)** Grand-averaged ErrPs. The shaded area around the line represents the standard deviation across all participants. The formats are the same as those in Figures 3A, B. **(C)** Classification result. The format is the same as that in Figure 4. ErrP, Error-related potentials.

Figure 5 summarizes the questionnaire responses, ErrP, and classification results for the first and last single-task sessions of 13 participants who underwent trials to assess the effect of temporal adaptations. The grand-averaged scores for the reliability of kinesthetic motor imagery were 2.82 for the first session and 3.23 for the last session (Figure 5A). Table 2 corresponds to Figure 5B and summarizes the peak amplitudes and latencies of the ERN and Pe. The single-trial ErrP classification accuracies were 68.6% and 69.6% for the first and last sessions, respectively (Figure 5C).

**Table 2:** Summary of peak amplitude and peak latency of ERN and Pe for 13 participants in the first and last sessions of the single-task scenario.

		**Peak amplitude (**μV**)**		**Peak latency (ms)**	
		**ERN**	**Pe**	**ERN**	**Pe**
First session	Erroneous	−4.49 ± 2.43	12.4 ± 4.46	224 ± 35.5	418 ± 26.2
	Correct	−2.62 ± 1.09	8.51 ± 2.95	201 ± 35.3	405 ± 50.7
Last session	Erroneous	−3.60 ± 2.11	12.0 ± 5.10	196 ± 32.3	403 ± 28.2
	Correct	−2.00 ± 0.906	6.99 ± 2.64	178 ± 19.7	371 ± 70.1

Values are presented as the mean ± standard deviation. ERN, error-related negativity; Pe, error positivity.

The results of the one-way repeated-measures ANOVA for the questionnaire showed no significant main effects, indicating that temporal factors did not influence the reliability of motor imagery in this experiment.

Regarding the peak amplitude of ERN, a significant main effect of the feedback type was observed (*F*_(1, 12)_ = 13.9, *p* < 0.05). Regarding the peak latency of ERN, significant main effects were observed for both sessions (*F*_(1, 12)_ = 23.2, *p* < 0.05) and the feedback type (*F*_(1, 12)_ = 5.18, *p* < 0.05). The peak amplitude of Pe showed a significant main effect of the feedback type (*F*_(1, 12)_ = 25.7, *p* < 0.05), whereas no significant main effects were observed for the peak latency of Pe. The findings indicated that temporal factors had a significant influence on the peak latency of ERN.

The ANOVA of ErrP classification accuracy revealed no significant main effects of temporal factors, indicating no influence on the classification performance.

## 4 Discussion

This study aimed to investigate the impact of sensorimotor multitasking on electrophysiological responses associated with interaction errors in a motor imagery-based BCI to aid the development of an ErrP-BCI suitable for real-life multitasking scenarios. Questionnaire analyses indicated that multitasking interfered with motor imagery. However, the peak amplitudes and latencies of both ERN and Pe remained unaffected by multitasking. Despite no significant changes in ErrPs, classification accuracy decreased owing to the influence of multitasking. The accuracy of ErrP classification significantly decreased when a single-task ErrP-BCI was applied to more difficult sensorimotor multitasking scenarios. The classification accuracy for all conditions exceeded the chance level, demonstrating that errors can still be detected by ErrP even in multitasking scenarios.

Over the past 20 years, various studies have explored how changes in attentional resources and cognitive load affect ErrP. Sleep deprivation has been demonstrated to slow reaction times and reduce Pe amplitude (Boardman et al., 2024). Moreover, ERN amplitude declines as sustained attention decreases (Xiao et al., 2015). Research on attentional bias correction has shown that diminished attentional focus leads to smaller ERN amplitudes (Nelson et al., 2017). In contrast, increased attention, as evaluated by task performance in the flanker task, enhances amplitudes of ERN, CRN, and Pe (Grützmann et al., 2014). These findings suggest that ErrPs are influenced by attentional resources and cognitive load. Tanaka et al. (2005) and Klawohn et al. (2016) observed that in multitasking scenarios, ERN amplitude decreased with an increase in cognitive load.

This study focused on multitasking in daily life and investigated the impact of simultaneous sensorimotor control and BCI operations on ErrPs. Based on previous studies on multitasking and ErrPs (Tanaka et al., 2005; Klawohn et al., 2016), we hypothesized that sensorimotor control would interfere with motor imagery and lead to changes in ErrP characteristics. However, contrary to our hypothesis, no changes in ERN or Pe owing to multitasking were observed. One possible explanation is that cognitive and attention loads imposed by the multitasking scenarios herein may not have been sufficient to alter ErrP. Tanaka et al. (2005) investigated changes in ErrPs with increasing cognitive load and reported significantly decreased ERN only at the highest difficulty level. This suggests that neural responses due to attentional resource distribution may not change continuously with increasing difficulty but rather exhibit discrete changes once certain thresholds are exceeded. Moreover, Klawohn et al. (2016) observed no significant changes in ErrPs between the two types of multitasking but noted a difference between the single-task and multitasking conditions, which further corroborates this hypothesis. Herein, the participants perceived the hard tasks as difficult (Figure 2A); however, no changes in ErrPs were observed, suggesting that ErrPs may remain stable even during challenging sensorimotor multitasking. This is a promising result for the future use of ErrP-BCIs as it implies that errors can be reliably detected even in complex scenarios.

**Figure 6:**
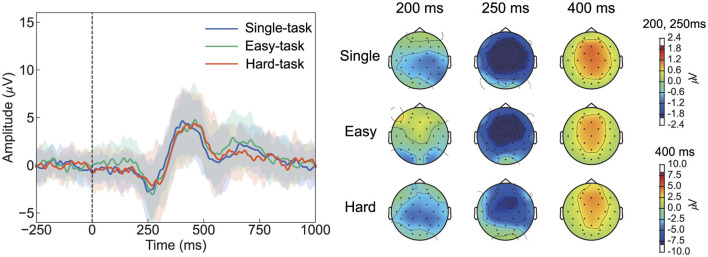
Differences in waveforms and topographic distribution maps at Cz, illustrating the differences in grand-averaged ErrPs between erroneous and correct feedback. The shaded area around the line represents the standard deviation across all participants. The formats are the same as those in Figures 3A, B. ErrP, Error-related potentials.

The ErrPs observed in this study exhibited reduced ERN amplitudes and lacked the characteristic positive peak preceding ERN, differing from typical interaction ErrPs (Figure 6). Typical interaction ErrPs are characterized by the difference in waveforms between erroneous and correct feedback, with an initial positive peak at approximately 200 ms after the feedback, followed by a large negative deflection at approximately 250 ms, and a third prominent positive peak at approximately 320 ms (Ferrez and Del R. Millan, 2008). These differences in ErrPs are consistent with previous findings of Si-Mohammed et al. (2020), who compared different types of error feedback in a virtual reality system. Therein, one type of error feedback occurred when a virtual object was suddenly lost during a task, thereby providing immediate and abrupt indications of failure. This type of feedback led to typical interaction ErrPs, characterized by a positive peak at approximately 200 ms, followed by a negative peak at 250 ms. Conversely, in another type of error feedback–termed gradual feedback–participants were informed of failure only after completing the task, despite having successfully transported the virtual object. Gradual feedback produced a different ErrP pattern compared with that of the immediate feedback, which was similar to that observed in Figure 5; it showed a small negative peak at approximately 150 ms, followed by a positive peak at 250 ms. Xavier Fidêncio et al. (2022) reviewed the differences in ErrPs and reported that in cases of continuous or gradual feedback, the clear ERN peak tended to diminish as the error occurred progressively. The ErrP waveform observed herein was consistent with previous findings regarding gradual feedback. Instead of responding to an immediate error, the participants may have processed the feedback more slowly as they monitored the cursor's movement. This gradual understanding of the error likely led to a smaller ERN peak compared with that of typical interaction ErrPs. Regarding ErrP classification, previous studies have reported that typical interaction ErrPs, with their more distinct features, result in higher classification accuracy than gradual error types (Si-Mohammed et al., 2020). These findings suggest that the feedback presentation method plays a critical role in accurately detecting errors in BCI systems.

This study revealed that ERN latency decreased over time (Figure 5, Table 2). A previous study investigating the effects of fatigue on the ERN reported that ERN amplitude was affected but no statistically significant changes in latency were observed (Xiao et al., 2015). Since no impact on amplitude was observed herein, the change in ERN latency was unlikely to be due to fatigue. The most plausible explanation for the observed decrease in ERN latency over time is habituation. Shortening of ERN latency has been demonstrated in studies comparing differences in auditory and visual feedback (Faßbender et al., 2023). Reportedly, auditory feedback leads to shorter ERN latencies compared with visual feedback (Miltner et al., 1997; Threadgill et al., 2020), providing evidence that the speed of error recognition varies depending on the type of feedback. Moreover, although the differences were not significant, the questionnaire results regarding the reliability of motor imagery showed slightly higher scores in the final session, further corroborating the hypothesis of habituation effects (Figure 5A). Overall, increased familiarity with the BCI operation potentially improved the speed of error recognition, which may have led to shorter ERN latencies during the final session.

We believe that the shortening of the ERN latency due to habituation did not affect the main objective of this study, which was to analyze ErrPs across different multitasking difficulty levels. Studies on ErrPs in multitasking scenarios with cognitive load have reported reductions in ERN amplitude but no changes in ERN latency (Tanaka et al., 2005; Klawohn et al., 2016). Similarly, reports on ErrP variations due to changes in attention have focused primarily on ERN amplitude, with no reports on ERN latency (Grützmann et al., 2014; Xiao et al., 2015; Nelson et al., 2017). The lack of change in ERN latency during sensorimotor multitasking observed herein is consistent with these previous findings.

This study has several limitations that should be addressed in future studies. First, the system was not tested in real-time; therefore, further investigation is required to evaluate its practicality for real-world applications. Although artifact removal was performed herein using cleaned EEG data, future studies should incorporate algorithms capable of real-time artifact removal. Second, errors due to sensorimotor control were excluded from the study. However, in practical scenarios, ErrPs may be triggered by a combination of errors in BCI operations and sensorimotor control. Further studies are needed to explore how these mixed errors affect ErrP classification. Third, in ErrP studies, errors are often intentionally introduced, which may lead to a credit assignment problem. However, this issue is seldom discussed in the literature. Similarly, in this study, the lack of a direct relationship between motor imagery and feedback may introduce a credit assignment problem. Future research may need to provide feedback that reflects motor imagery EEG decoding to verify whether our conclusions hold in real-world scenarios. Finaly, although the sample size of 25 participants is consistent with prior studies on BCI and ErrPs (Penaloza and Nishio, 2018; Huang et al., 2020; Usama et al., 2020; Iwane et al., 2023), the limited number of participants–particularly the underrepresentation of female participants–may affect the generalizability of our findings. This study represents a foundational step in investigating how multitasking involving sensorimotor control influences ErrPs during BCI operation. However, further research with larger and more diverse participant groups is necessary to validate these findings and ensure their broader applicability across different populations.

## Data Availability

The raw data supporting the conclusions of this article will be made available by the authors, without undue reservation.

## Footnotes

^1^ https://github.com/sccn/labstreaminglayer
