# Supplementary material for: Error-related potentials during multitasking involving sensorimotor control: an ERP and offline decoding study for brain-computer interface
Source: Front Hum Neurosci. 2025 Jan 28;19:1516721. doi: 10.3389/fnhum.2025.1516721 (PMC11810888; doi:10.3389/fnhum.2025.1516721)
Supplement: Supplementary file 1 [file Table_1.docx]

Supplementary Material

**Supplementary Table 1.** Summary of the two-way repeated measures ANOVA with factor as Feedback (Correct vs. Erroneous) and Scenario (single-, easy-, and hard-task).

|  |  | Amplitude | | Latency | |
| --- | --- | --- | --- | --- | --- |
|  |  | ERN | Pe | ERN | Pe |
| Feedback | F(1,24) | 26.4 | 72.2 | 8.35 | 13.5 |
|  | p | **2.95 × 10^-5^** | **1.07 × 10^-8^** | **0.008** | **0.001** |
|  | η² | 0.155 | 0.221 | 0.060 | 0.072 |
| Scenario | F(1,24) | 0.423 | 0.083 | 0.377 | 0.160 |
|  | p | 0.658 | 0.921 | 0.688 | 0.853 |
|  | η² | 3.95 × 10^-3^ | 4.34 × 10^-4^ | 4.25 × 10^-3^ | 2.14 × 10^-3^ |
| Feedback × Scenario | F(1,24) | 1.08 | 0.477 | 1.13 | 0.311 |
|  | p | 0.347 | 0.623 | 0.332 | 0.734 |
|  | η² | 6.52 × 10^-3^ | 9.94 × 10^-4^ | 9.35 x 10^-3^ | 2.21 × 10^-3^ |

ERN: error-related negativity, Pe: error positivity.
